# Evaluating mineral biomarkers as mediators and moderators of behavioural improvements in a randomised controlled trial of multinutrients for children with attention-deficit/hyperactivity disorder

**DOI:** 10.1017/S0007114524001132

**Published:** 2024-08-14

**Authors:** Lisa M. Robinette, Jeanette M. Johnstone, Priya Srikanth, Alisha M. Bruton, Martina Ralle, Hayleigh K. Ast, Ryan D. Bradley, Brenda Leung, L. Eugene Arnold, Irene E. Hatsu

**Affiliations:** 1 Department of Human Sciences, The Ohio State University, Columbus, OH, USA; 2 Center for Mental Health Innovation, Department of Psychiatry, Oregon Health & Science University, Portland, OR, USA; 3 National University of Natural Medicine, Helfgott Research Institute, Portland, OR, USA; 4 Oregon Health & Science University, Portland, OR, USA; 5 Department of Molecular and Medical Genetics, Oregon Health & Science University, Portland, OR, USA; 6 Herbert Wertheim School of Public Health, University of California San Diego, San Diego, CA, USA; 7 Faculty of Health Sciences, University of Lethbridge, Lethbridge, AB, Canada; 8 Department of Psychiatry & Behavioral Health, The Ohio State University, Columbus, OH, USA; 9 OSU Extension, The Ohio State University, Columbus, OH, USA

**Keywords:** Attention-deficit/hyperactivity disorder, Minerals, Mediation, Moderation, Children, Emotional dysregulation, Supplement, Nutrients

## Abstract

Essential minerals are cofactors for synthesis of neurotransmitters supporting cognition and mood. An 8-week fully-blind randomised controlled trial of multinutrients for attention-deficit/hyperactivity disorder (ADHD) demonstrated three times as many children (age 6–12) had significantly improved behaviour (‘treatment responders’) on multinutrients (54 %) compared with placebo (18 %). The aim of this secondary study was to evaluate changes in fasted plasma and urinary mineral concentrations following the intervention and their role as mediators and moderators of treatment response. Fourteen essential or trace minerals were measured in plasma and/or urine at baseline and week eight from eighty-six participants (forty-nine multinutrients, thirty-seven placebos). Two-sample *t* tests/Mann–Whitney *U* tests compared 8-week change between treatment and placebo groups, which were also evaluated as potential mediators. Baseline levels were evaluated as potential moderators, using logistic regression models with clinical treatment response as the outcome. After 8 weeks, plasma boron, Cr (in females only), Li, Mo, Se and vanadium and urinary iodine, Li and Se increased more with multinutrients than placebo, while plasma phosphorus decreased. These changes did not mediate treatment response. However, baseline urinary Li trended towards moderation: participants with lower baseline urinary Li were more likely to respond to multinutrients (*P* = 0·058). Additionally, participants with higher baseline Fe were more likely to be treatment responders regardless of the treatment group (*P* = 0·036.) These results show that multinutrient treatment response among children with ADHD is independent of their baseline plasma mineral levels, while baseline urinary Li levels show potential as a non-invasive biomarker of treatment response requiring further study.

Attention-deficit/hyperactivity disorder (ADHD) is a common neurodevelopmental disorder affecting up to 10 % of children in the USA with an estimated annual societal cost of $124·5 billion in the USA alone^(1,2)^. Nutritional therapies, including healthy diets and nutrient supplementation, are emerging as ADHD treatment modalities with the potential for minimal adverse and long-term side effects compared with standard pharmacological treatment^(3)^. Although the underlying mechanisms through which nutritional interventions function to improve ADHD symptoms remains unclear, they likely address deficiencies in essential minerals possibly related to genetic need for greater intake. A heterogeneous group of studies and meta-analyses have examined mineral status in ADHD with evidence of lower levels of Fe, Zn and Mg in biological samples (e.g. blood, hair and urine) in ADHD patients compared with controls, and several placebo-controlled trials support the efficacy of mineral supplementation (either as single nutrients or in combination with other nutrients) to improve ADHD symptoms^(4–10)^. Minerals are critical for the synthesis of serotonin and catecholamine neurotransmitters (dopamine and norepinephrine), among many other roles^(11,12)^. For example, Fe and Cu play key roles in the brain as enzyme cofactors in neurotransmitter synthesis (e.g. Fe as a co-factor for tyrosine hydroxylase and tryptophan hydroxylase for synthesis of dopamine/norepinephrine and serotonin, respectively, and Cu as a co-factor for dopamine beta-hydroxylase for the conversion of dopamine to norephinephrine). Additionally, Mg and Zn function as inhibitors and blockers of key neurotransmitter receptors and transporters (e.g. Zn with dopamine transporter and Mg and Zn with the N-methyl-D-aspartate glutamate receptor) (Fig. 1)^(4,9,13–17)^.

**Fig. 1.**
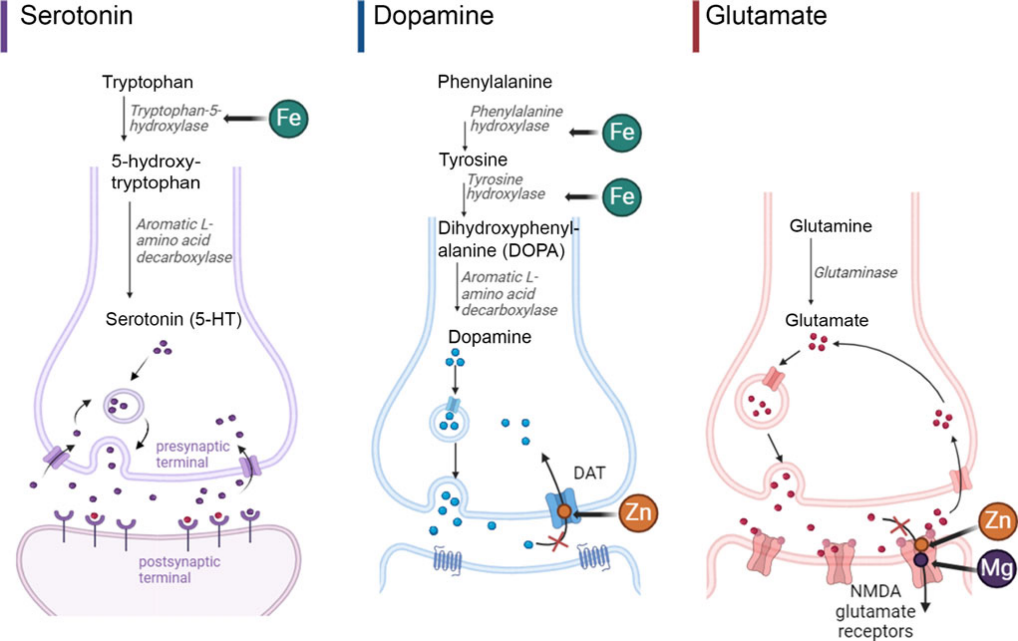
Representative essential minerals’ role in serotonin, dopamine, and glutamate synthesis and neurotransmission. Adapted from ‘Glutamate Synthesis and Cycling’ and ‘Mechanism of action of Selective Serotonin Reuptake Inhibitors’ by BioRender.com (2024). Retrieved from https://app.biorender.com/biorender-templates.

These analyses utilised data from a fully blinded randomised controlled trail (RCT) of children with ADHD and emotional dysregulation that showed benefit for broad-spectrum multinutrient supplementation (referred to hereafter as ‘multinutrients’) over placebo in the primary outcome of clinician-rated global improvement in functioning and behaviour (referred to hereafter as ‘treatment response’)^(18)^. Mediation and moderation analyses in RCT are crucial to identify for whom treatments work and to inform hypotheses about why they work^(19)^. The multinutrients used in the RCT contained fourteen essential and trace minerals that were measured in plasma and/or urine: boron (B), Cr, Cu, Fe, Li, iodine (I), Mg, Mn, Mo, Ni, phosphorus (P), Se, vanadium (V) and Zn^(18)^. The first aim of these analyses was to measure changes in plasma and urinary mineral concentrations following an 8-week intervention using multinutrients in children with ADHD and emotional dysregulation, with the hypothesis that mineral concentrations will significantly increase in the multinutrient group compared with the placebo group. The second aim was to determine whether mineral concentrations moderate or mediate behavioural improvements, with the hypotheses that low baseline mineral concentrations will moderate, and minerals that increased after 8 weeks of multinutrients will mediate behavioural improvements.

## Methods

### Trial design and participants

This is a secondary data analysis using biological samples collected from the Micronutrients for ADHD in Youth Study. The primary outcomes of behavioural improvements and safety profile, along with details of study design are published elsewhere^(18,20,21)^. Briefly, the Micronutrients for ADHD in Youth RCT was an 8-week randomised fully blind placebo-controlled trial that examined the efficacy of a multi-vitamin/mineral supplement (‘multinutrients’) as treatment for children with ADHD and emotional dysregulation. Participants included in the study were age 6–12 years and had 6 or more inattention and/or hyperactivity/impulsivity symptoms on the parent-reported Child and Adolescent Symptom Inventory-5^(22,23)^. Additionally, participants demonstrated at least one symptom of irritability or anger from the Child and Adolescent Symptom Inventory-5 Oppositional Defiant Disorder or Disruptive Mood Dysregulation Disorder subscales. Additional inclusion criteria included being psychotropic-medication free for at least 2 weeks prior to the baseline assessment, willing/able to swallow 9–12 capsules per day, and willing to give blood samples at baseline and week 8 visits. Exclusion criteria included any neurological disorder (e.g. intellectual disability, autism spectrum disorder) or other major psychiatric conditions requiring hospitalisation (e.g. significant mood disorder, active suicidal ideation); any serious medical condition such as diabetes, hyperthyroidism, inflammatory bowel disease; and any known abnormality of mineral metabolism (e.g. Wilson disease, hemochromatosis). Participants were recruited from three sites: two in the USA (Columbus, OH and Portland, OR) and one in Canada (Lethbridge, Alberta). The Micronutrients for ADHD in Youth RCT was conducted according to the guidelines laid down in the Declaration of Helsinki and all procedures involving human subjects/patients were approved by Institutional Review Boards (IRB) at The Ohio State University (OSU; IRB # 2017H0188) and Oregon Health & Science University (OHSU; IRB # 16870) and the Conjoint Health Research Ethics Board at University of Calgary (REB# 17-0325) for the University of Lethbridge. The RCT was approved by the USA Food and Drug Administration (FDA) under an investigational new drug application (IND #127832) and by Health Canada (Control #207742) and was prospectively registered in the ClinicalTrials.gov database (NCT #03252522). At the baseline visits, written informed consent was obtained from all parents/guardians and assent from children prior to any study procedures.

### Intervention

**Fig. 2.**
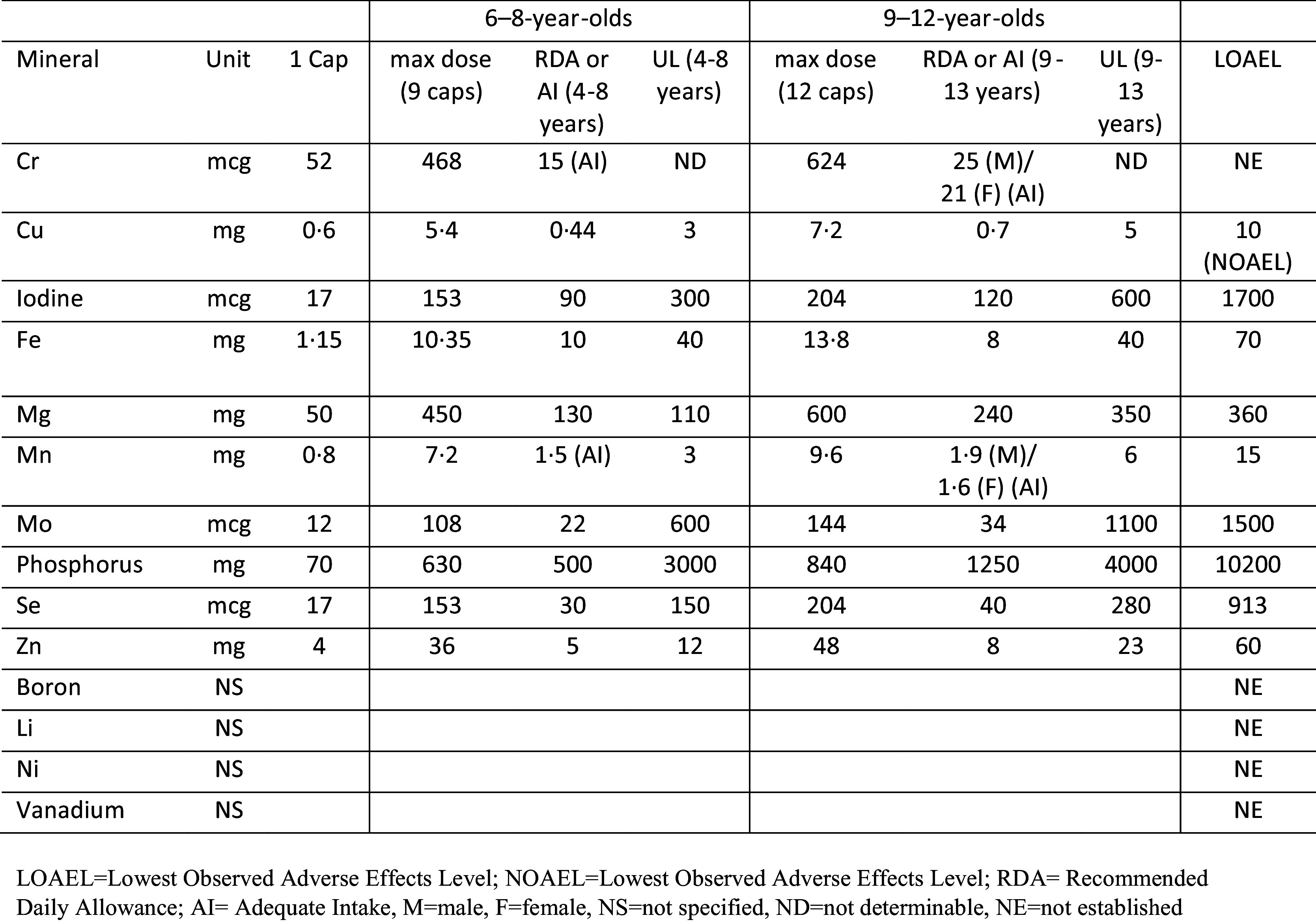
Dosage of each mineral contained in multinutrient formulation and RDA/AI, UL, and LOAEL.

The multinutrient intervention consisted of a blend of all known vitamins and essential minerals, plus amino acids, and antioxidants (full list of ingredients in the formula is available in online Supplementary Materials). A dose of 9–12 capsules per day provided nutrient dosages generally above the recommended dietary allowance (RDA), but below the lowest observed adverse effects level except for two above the lowest observed adverse effects level (Mg and niacin (B3)). Seven other nutrients were above the upper tolerable intake level (copper, Mn, Se, zinc, vitamin A (retinyl palmitate), pyridoxine (B6), and folate), but below lowest observed adverse effects level. The dosage, RDA, and upper tolerable intake level of each mineral are shown in Fig. 2 and further discussion of dosage and rationale has been published^(22)^. The placebo capsules, which looked identical to the multinutrient capsules, contained cellulose filler and 0·1 mg of riboflavin per capsule (total dose 0·9–1·2 mg/d, which is above RDA for this age group) added to mimic urine colour when an individual is supplemented with B vitamins. Hardy Nutritionals (Raymond, AB, Canada) provided the active intervention formula, Daily Essential Nutrients, and the placebo capsules without cost, but had no role in the study design, data collection and analysis, or interpretation of the results. Treatment adherence was monitored from the number of returned pills at each visit, which were counted by research staff not associated with the study. No dietary education, advice, or guidelines were provided as part of the intervention.

### Biological samples

To meet FDA requirements, participants at the 2 USA sites (OH and OR) were required to provide fasted blood and urine samples in the morning at their baseline and week 8 on-site study visits to identify contraindications or subsequent adverse events by monitoring complete blood count, comprehensive metabolic panel, thyroid panel, and urinalysis. Safety tests were not required by Health Canada; therefore, blood and urine samples were not collected at that site. At the time of safety blood draws, additional quantities of blood (maximum of 23 ml of blood drawn per visit) and urine were collected and frozen for future mechanistic analyses. Blood samples were processed and separated into plasma and red blood cell portions using standard methods. They were then aliquoted and stored at −80°C. For urine samples, participants were provided with a sterile urine specimen collection container and instructed to fill it at least halfway full (4 ml minimum). The urine samples were aliquoted and stored at −80°C until analysis.

### Sample size

**Fig. 3.**
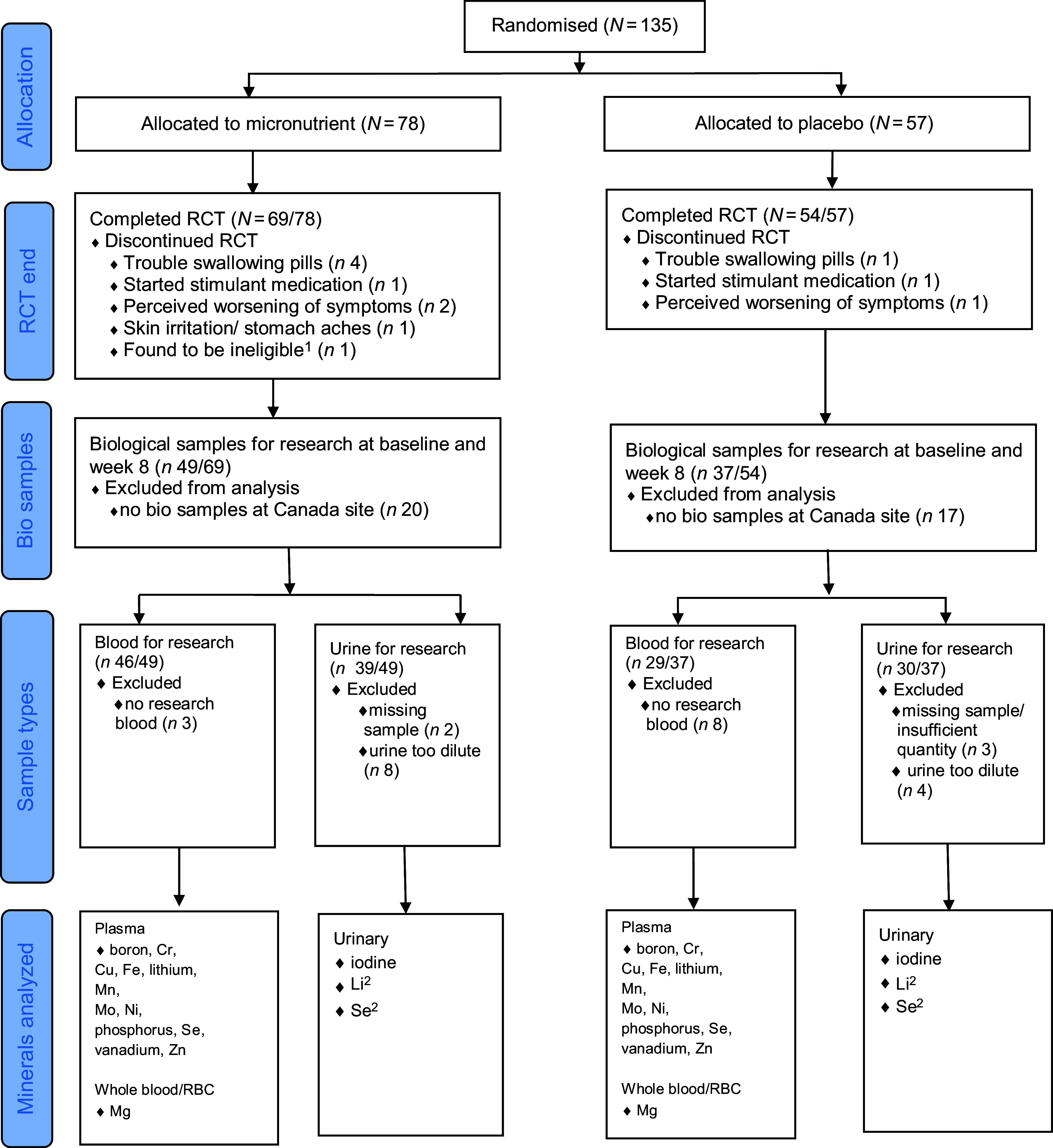
CONSORT flow diagram for MADDY RCT updated to include biological samples used in these analyses. ^1^Participant met ADHD symptom scores criteria at initial screening, but no longer met required scores at baseline assessment. ^2^Referred to as Li(urine) and Se(urine) to differentiate from Li and Se measured in plasma from research blood.

For the RCT design, a sample size of 123 was needed to detect differences between groups using a 3:2 randomisation ratio of multinutrients to placebo. 135 participants were recruited to allow for attrition, of which 123 completed the RCT (attended week 8 visit)^(18)^. Frozen blood and urine samples were available for (*n* 86) participants from the two USA study sites (see Fig. 3). Specifically, 75 participants had plasma samples (46 multinutrients, 29 placebo), and 69 participants had urine samples (39 multinutrients, 30 placebo) (Fig. 3). Missing samples were primarily due to insufficient quantity of blood drawn during the visit for research purposes, and/or collected urine that was too dilute to measure minerals.

### Measures

#### Sociodemographic and anthropometric measures

At the baseline visit, demographic information was collected including parent-reported questions on gender, ethnicity, race, parent/guardians’ level of education, occupation, and family income. At each study visit, body mass index (BMI) was calculated based on height, measured using a stadiometer with adjustable headpiece, and weight, measured using a calibrated digital scale.

#### Treatment response measures

Improvement in behaviour and overall functioning (i.e. treatment response) was rated at week 8 using the blinded-clinician-rated Clinical Global Impressions-Improvement (CGI-I) scale, an *a priori* primary outcome. CGI-I assesses symptom improvement or worsening at week 8 compared with baseline (rated from 1 = very much improved to 7 = very much worse, with 4 = no change)^(24)^. Trained study staff used all available data, including behavioural questionnaires, interviews with parent and child at each visit, in-clinic observations, to rate the CGI-I at week 8. If CGI-I data were missing at week 8, last observation carried forward from week 4 was used. The primary outcome measure was a dichotomous ‘treatment responder’ (defined by a rating of 1 or 2 (‘very much improved’ or ‘much improved’) on the CGI-I scale at week 8) or ‘treatment non-responder’ (defined by a rating of 3 (somewhat improved) to 7 (very much worse)). To standardise ratings among sites, all CGIs were reviewed at weekly cross-site video calls with blinded senior study staff, including doctoral-level clinicians.

#### Mineral concentrations

For most minerals, adequacy status is typically determined from blood concentrations. When available in excess, however, almost all minerals are excreted via urine. As such, urinary mineral concentrations could indicate exposure or demand for minerals as well as indicate disease status^(25,26)^. This study measured the concentrations of fourteen different minerals contained in the multinutrient formula in plasma and/or urine: thirteen minerals from plasma samples (B, Cu, Cr, Fe, Li, Mg, Mn, Mo, Ni, P, Se, V, and Zn), of which Li and Se were also measured in urine samples, along with I (see Fig. 3)

#### Plasma mineral concentrations

Plasma samples were measured for the 13 minerals in the OHSU Elemental Analysis Shared Resource using inductively coupled plasma mass spectroscopy performed on an Agilent 7700× equipped with an ASX 500 autosampler. The above listed minerals were measured in plasma, except Mg which was measured in whole blood (WB) at OR site or red blood cells (RBC) at OH site (referred to hereafter as Mg (WB) and Mg (RBC), respectively, to differentiate from plasma samples). The system was operated at a radio frequency power of 1550 W, an argon plasma gas flow rate of 15 l/min, argon carrier gas flow rate of 0·9 l/min. Elements were measured in no-gas mode, kinetic energy discrimination (KED) mode using helium gas (4·3 ml/min), or in mass-on-mass (hydrogen) mode using hydrogen gas (3·4 ml/min). Data were quantified from serial dilutions by weight and volume of calibration standards for each element using an 11-point calibration curve. For each sample, data were acquired in triplicates and averaged. A coefficient of variance (CoV) was determined from frequent measurements of a sample containing 5–10 ppb of the elements to be analyzed; this is summarised as a mean and range of CoV for each mineral in online Supplementary Materials. An internal standard (scandium, germanium, bismuth) continuously introduced with the sample was used to correct for detector fluctuations and to monitor plasma stability.

#### Urinary mineral concentrations

The urine sample data were generated by ZRT Laboratory (Beaverton, OR) using inductively coupled plasma mass spectroscopy. Laboratory staff were blinded to participants’ treatment group allocation. Frozen urine was sent to ZRT Laboratory on dry ice and stored frozen at −80°C until all samples were collected, then processed for testing. Urine was analyzed for I, Li, and Se (referred to hereafter as I(urine), Li(urine) and Se(urine) to differentiate from plasma inductively coupled plasma mass spectroscopy results) as part of the ‘Toxic and Essential Elements panel’ from ZRT Laboratories. Results were reported in micrograms per gram (μg/g) of creatinine to correct for urine dilution. Urine was thawed, mixed, and 1 ml was transferred to acid-treated Whatman 903 filter strips and dried overnight at room temperature, then stored at −80°C in plastic bags with desiccant. Within- and between-assay precision for each of these measures are included in online Supplemental Materials.

#### Estimated mineral intake

Baseline dietary intake was assessed by a parent or caregiver using the Vioscreen^TM^ food frequency questionnaire within 3 d of the baseline visit (https://www.viocare.com/vioscreen.html; Viocare Inc, Kingston, NJ). Vioscreen^TM^ is a validated, graphics-based dietary analysis software that provides the equivalent of 90 d of nutrition tracking in about 20 min^(27)^. It uses food and nutrient information from the Nutrition Coordinating Center Food and Nutrient Database (University of Minnesota Division of Epidemiology and Community Health in Minneapolis) to calculate estimated nutrient intake. Estimated intakes were calculated for Cu, Fe, Mg, Mn, P, Se, and Zn.

### Statistical methods

Baseline demographic and biometric characteristics (age, sex, race, ethnicity, family income level and BMI) were reported as mean and sd for parametric data and median and interquartile range for non-parametric data. Categorical variables were reported as frequency (percent). Characteristics were calculated overall and compared between intervention arms using two-sample *t* tests with equal variances (or unequal variance, if ratio of standard deviations > 2) for continuous variables and the Pearson *χ* ^2^ or Fisher’s exact test (if expected counts < 5) for categorical variables. To confirm that the findings from the RCT still hold in this subset of participants, frequency of responders and non-responders between intervention arms was checked using Pearson’s *χ* ^2^ test.

Any mineral that was below detection limits was coded as half of the detection limit for data analysis^(28)^. Mg was evaluated separately for each site because different blood sample types were used (whole blood *v*. red blood cells). Boron was analysed for OR site only since most values at OH site were below detection limits. To evaluate for potential confounding factors, site, age, sex, BMI and estimated nutrient intakes were evaluated for associations with each mineral concentration. For parametric data, Pearson correlation coefficients were used for continuous variables and two-sample *t* test to compare across categorical variables. For non-parametric data, Spearman correlation coefficients were used for continuous variables and Mann–Whitney *U* test for categorical variables with two categories.

For each mineral, change after 8 weeks of supplementation was calculated for each participant as a percent change using the value at week 8 minus the value at baseline divided by baseline value × 100 (referred to hereafter as ‘8-week percent change’). To determine if the 8-week percent change for each mineral was different between the multinutrient and placebo group, two-sample *t* test or Mann Whitney *U* test was used.

To test for mediation and moderation, logistic regression models were used comparing the multinutrient group to the placebo group, with independent variables of intervention arm (I), the potential mediator/moderator (M) and the multiplicative interaction variable (i.e. IxM) using methodology recommended by Kraemer et al. (2002)^(19)^. The outcome measure was CGI-I at week 8 as a dichotomous variable (responder *v*. non-responder) using logistic regression models to estimate the OR and 95 % CI.

Baseline mineral concentrations were evaluated as potential moderators while any minerals with significant 8-week percent change between multinutrient and the placebo group were evaluated as potential mediators. First, a 3-way interaction of site-by-treatment group-by-potential moderator/mediator was tested to determine if there were significant site interactions. If the 3-way interaction was not significant, the model included the potential moderator/mediator in an interaction term with intervention arm, plus the individual terms for the potential moderator/mediator, intervention arm, and site, sex, age, and BMI entered as covariates. Any significant interactions were probed to determine direction of moderation/mediation.

Statistical significance was defined as a two-sided *P* value < 0·05. Given the exploratory nature of these analyses, we did not correct for multiple testing. All analyses were conducted using Stata version 18 software (StataCorp LLC)^(29)^.

## Results

### Study population characteristics

Socio-demographic characteristics and baseline nutrient levels of the study participants with blood and/or urine samples at baseline and week 8 (*n* 86) are shown in Table 1. This sample was 62 % male, predominantly of White race, non-Hispanic, with median (interquartile range) age in years 10·2 (8·6, 11·1). Socio-demographic and biometric characteristics were similar between the multinutrient and placebo groups (Table 1). The primary finding of the RCT that a significantly higher portion of participants in the multinutrient group were responders compared with the placebo group remained consistent in this subset with four times as many treatment responders in the multinutrient group (57·2 % responders) than the placebo group (13·5 % responders) (*P* < 0·001).

**Table 1.** Characteristics of the study population comparing multinutrient and placebo groups (Numbers and percentages; median values and interquartile ranges; mean values and standard deviations)

	Total (*n* 86)		Multinutrient (*n* 49)		Placebo (*n* 37)		
Characteristics	Median	IQR	Median	IQR	Median	IQR	*P* ||
Child’s age (in years), median (IQR)	10·2	8·6, 11·1	10·3	8·6, 11·3	10·0	8·8, 11·1	0·428
BMI, median (IQR)	16·6	15·4, 18·6	16·8	15·5, 18·7	16·0	15·3, 18·6	0·328
	*n*	%	*n*	%	*n*	%	
Child’s sex, *n* (%)							0·074
Male	62	72·1	39	79·6	23	62·2	
Female	24	27·9	10	20·41	14	37·84	
Family income, annual USD, *n* (%)							0·367
< $30 000	7	8·1	5	10·2	2	5·4	
$30 001–60 000	14	16·3	6	12·2	8	21·6	
$60 001–80 000	9	10·5	7	14·3	2	5·4	
> $80 001	56	65·1	31	63·3	25	67·6	
Parent marital status, *n* (%)							0·148
Married	66	76·7	35	71·4	31	83·8	
Divorced	15	17·4	9	18·4	6	16·2	
Single	5	5·8	5	10·2	0	0·0	
Parent educational level, *n* (%)							1·000
High school	8	9·3	5	10·2	3	8·1	
Technical college/trade school	18	20·9	10	20·4	8	21·6	
University or higher	60	69·77	34	69·4	26	70·3	
Ethnicity*, *n* (%)							0·376
Not Hispanic or Latino	58	67·4	30	61·2	28	75·7	
Hispanic or Latino	7	8·1	6	12·2	1	2·7	
Other**	3	3·5	2	4·1	1	2·70	
Race*, *n* (%)							0·257
Asian	4	4·7	4	8·2	0	0·0	
Black	8	9·3	3	6·1	5	13·5	
White	70	81·4	39	79·6	31	83·8	
Other††	1	1·16	1	2·0	0	0	
Baseline plasma mineral levels	Total (*n* 75)		Multinutrient (*n* 46)		Placebo (*n* 35)		*P* ¶
	Median	IQR	Median	IQR	Median	IQR	
Boron†, ug/l							
Mean	28·9		29·7		27·6		0·592
sd	11·0		10·2		12·5		
Cr, ug/l, median (IQR)	0·97	0·63, 1·82	0·95	0·63, 1·95	1·04	0·68, 1·59	0·929
Cu, ug/l							
Mean	904·6		917·2		884·6		0·397
sd	161·2		162·6		159·8		
Fe, ug/l, median (IQR)	1157	841, 1402	1227	972, 1393	1124	756, 1430	0·252
Li, ug/l, median (IQR)	1·24	1·00, 1·55	1·27	1·06, 1·67	1·19	0·83, 1·44	0·228
Mg (WB)†, mg/l, median (IQR)	31·27	29·33, 33·64	31·38	29·71, 33·64	30·18	28·47, 33·80	0·412
Mg (RBC)‡, mg/l							
Mean	45·4		45·0		46·1		0·505
sd	4·6		5·0		4·0		
Mn, ug/l, median (IQR)	0·63	0·47, 0·81	0·67	0·49, 0·86	0·59	0·43, 0·71	0·045
Mo, ug/l							
Mean	1·2		1·2		1·1		0·075
sd	0·3		0·3		0·2		
Ni, ug/l, median (IQR)	1·34	0·73, 1·85	1·47	0·78, 1·94	1·07	0·61, 1·71	0·11
Phosphorus, mg/l, median (IQR)	153	140, 186	153	141, 182	157	138, 186	0·735
Se, ug/l							
Mean	191·8		190·3		194·1		0·499
sd	23·3		24·4		21·6		
Vanadium, ug/l, median (IQR)	0·93	0·19, 1·85	0·66	0·22, 1·98	1·12	0·19, 1·70	0·899
Zn, ug/l, median (IQR)	777·0	728·0, 848·0	776·5	728·0, 848·0	777·0	728·0, 843·0	0·946
Baseline urinary mineral levels	Total (*n* 69)		Multinutrient (*n* 39)		Placebo (*n* 30)		*P* ¶
Iodine, ug/g, median (IQR)	175·0	125·0, 247·0	175·0	120·0, 216·0	180·5	140·0, 288·0	0·637
Li§, ug/g, median (IQR)	27·0	21·0, 41·0	25·0	18·0, 40·0	29·0	22·0, 41·0	0·504
Se, ug/g, median (IQR)	88·0	72·0, 116·0	86·0	65·0, 116·0	92·0	74·0, 114·0	0·676

IQR, interquartile range; HEI-2015, Healthy Eating Index-2015.

Values are presented as medians (IQR) or means (sd) where noted for continuous variables or as frequencies (percentages) for categorical variables.

* 18 participants did not report ethnicity and three participants did not report race.

† Oregon participants only (*n* 37: 23 multinutrient, 14 placebo).

‡ Ohio participants only (*n* 38: 23 multinutrient, 15 placebo).

§ Results missing for 14 urinary samples for lithium (*n* 55: 30 multinutrient, 25 placebo).

|| *P* values comparing multinutrient to placebo groups using two-sample *t* tests with equal variances (or unequal variance, if ratio of standard deviations > 2) for continuous variables and the Pearson chi squared or Fisher’s exact test (if expected counts < 5) for categorical variables.

¶ *P* values comparing multinutrient to placebo groups for baseline minerals calculated using *t* test for parametric and Mann–Whitney *U* test for non-parametric variables.

** Includes Jewish, Japanese or other for ethnicity.

†† Includes American Indian/Native American or Alaska Native, Métis, Native Hawaiian/Pacific Islander or other for Race.

### Characterisation of baseline mineral concentrations

Baseline plasma and urinary minerals concentrations did not differ between intervention arms except for Mn (Table 1). All baseline plasma mineral concentrations, but not urinary minerals, differed by site (online Supplementary Table 1). Baseline Cr, Cu and Ni were significantly different by sex with all three minerals higher in males *v*. females (data shown as median (interquartile range) or mean (sd) for males *v*. females, *P* value): Cr (1·24 (0·73–2·39) *v*. 0·66 (0·52–0·97) ug/l, *P* = 0·001); Cu (931·81 (161·14) *v*. 839·05 (144·57) ug/l, *P* = 0·022); Ni (1·49 (1·01–2·01) *v*. 0·97 (0·56–1·42) ug/l, *P* = 0·012) (online Supplementary Table 1).

Child’s age was negatively correlated with baseline Mo, I and Se(urine), and BMI was negatively correlated with baseline Li, Mn, Mo and Li(urine) (online Supplementary Table 2). None of the baseline mineral concentrations correlated significantly with estimated mineral intake from the Vioscreen^TM^ FFQ, although urinary Se trended towards significant correlation with estimated Se intake (*r* = 0·23, *P* = 0·055) (online Supplementary Table 2).

### 8-week percent change in mineral concentrations

**Fig. 4.**
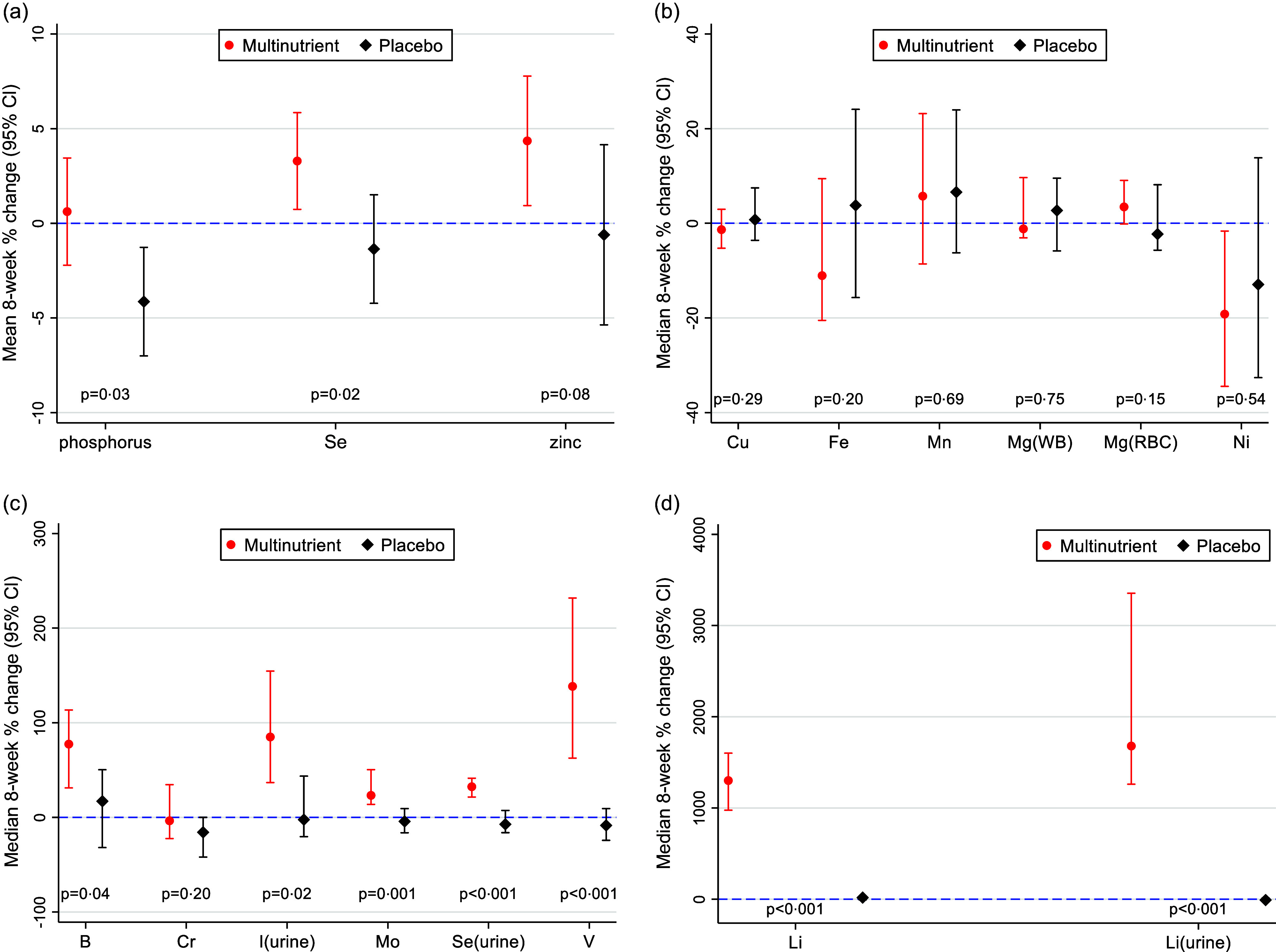
Eight-week percent change and 95 % CI for each mineral by treatment group. a. Mean 8-week % change and 95 % CI for parametric distributions (P, Se, and Zn); *P* values calculated with *t* test comparing multinutrient with placebo group. b. Median 8-week % change and 95 % CI for non-parametric distributions (Cu, Fe, Mn, Mg(WB), Mg(RBC), Ni); *P*-values calculated with Mann–Whitney *U* test comparing multinutrient to placebo group. c. Median 8-week percent change and 95 % CI for non-parametric distributions (B, Cr, I(urine), Mo, Se(urine), V); *P*-values calculated with Mann–Whitney *U* test comparing multinutrient to placebo group. c. Median 8-week percent change and 95 % CI for non-parametric distributions (Li, Li(urine)); *P*-values calculated with Mann–Whitney *U* test comparing multinutrient to placebo group.

As illustrated in Table 2 and Fig. 4, five plasma minerals (B, Li, Mo, Se and V) increased significantly in multinutrient group compared with placebo after 8 weeks and Zn trended towards significance. Phosphorus decreased in the placebo group compared with multinutrient after 8 weeks leading to a significant between-group change. Eight-week percent change for each plasma mineral was comparable between sites, except for Fe, Li and V (online Supplementary Table 3). Meanwhile, all three urinary minerals (I, Li and Se) had significant increases in the multinutrient group compared with placebo group after 8 weeks (Table 2 and Fig. 4). Eight-week percent change for each urinary mineral was comparable between sites (online Supplementary Table 3).

**Table 2.** 8-week percent change in mineral concentrations in multinutrient compared with placebo group (Median values and interquartile ranges)

	Multinutrient						Placebo						Between group 8-week % change
	Baseline		Week 8		Within group 8-week % change		Baseline		Week 8		Within group 8-week % change		
Mineral	Median	IQR	Median	IQR	Median	IQR	Median	IQR	Median	IQR	Median	IQR	*P*
Plasma													
Boron†, ug/l	30·0	18·0, 35·0	51·0	42·0, 67·0	77·4	26·3, 163·6	24·5	20·0, 35·0	29·0	14·0, 39·0	17·1	–29·5, 50·0	0·010
Cr, ug/l	1·0	0·6, 2·0	1·0	0·8, 1·7	–3·6	–38·5, 77·8	1·0	0·7, 1·6	0·7	0·5, 1·7	–15·7	–46·5, 6·3	0·196
Cu, ug/l	898·0	834·0, 1010·0	898·5	803·0, 999·0	–1·3	–7·8, 7·4	872·0	744·0, 996·0	894·0	784·0, 1034·0	0·8	–4·6, 10·5	0·290
Fe, ug/l	1226	972·0, 1393	988·5	898·0, 1212	–11·1	–28·2, 22·3	1124	756·0, 1430	1173	853·0, 1372	3·8	–18·4, 30·3	0·199
Li, ug/l	1·3	1·1, 1·7	19·4	13·4, 27·0	1301	700·8, 1975	1·2	0·8, 1·4	1·1	1·0, 1·8	17·9	–9·4, 40·0	< 0·001
Mg (WB)†, mg/l	31·4	29·7, 33·6	32·9	29·0, 36·0	–1·2	–3·7, 10·1	30·2	28·5, 33·8	30·3	29·6, 31·5	2·7	–3·8, 9·2	0·749
Mg (RBC)‡, mg/l	45·8	41·5, 49·4	48·1	39·9, 52·0	3·5	–1·5, 11·4	46·1	42·8, 48·0	45·6	40·2, 50·4	–2·3	–5·8, 8·5	0·153
Mn, ug/l	0·7	0·5, 0·9	0·7	0·5, 0·9	5·7	–20·6, 32·8	0·6	0·4, 0·7	0·6	0·5, 0·8	6·6	–17·7, 68·2	0·693
Mo, ug/l	1·2	1·0, 1·4	1·6	1·2, 2·0	23·3	2·3, 87·5	1·1	0·9, 1·2	1·1	0·9, 1·3	–4·3	–22·1, 16·3	0·001
Ni, ug/l	1·5	0·8, 1·9	1·1	0·8, 1·7	–19·2	–57·6, 59·8	1·1	0·6, 1·7	0·9	0·6, 1·3	–12·9	–33·6, 20·5	0·541
Phosphorus, mg/l	153·0	141·0, 182·0	157·0	141·0, 177·0	0·6	–5·9, 5·6	157·0	138·0, 186·0	149·0	137·0, 171·0	–3·8	–8·4, 0·0	0·026*
Se, ug/l	187·1	170·8, 209·1	195·0	178·0, 213·0	3·7	–0·5, 9·1	192·5	181·6, 205·6	189·0	180·0, 203·0	–3·1	–6·6, 3·4	0·020*
Vanadium, ug/l	0·7	0·2, 2·0	1·9	0·9, 2·9	138·4	46·5, 313·1	1·1	0·2, 1·7	1·1	0·2, 1·8	–8·5	–28·7, 23·0	< 0·001
Zn, ug/l	776·5	728·0, 848·0	791·0	753·0, 891·0	3·6	–4·7, 11·5	777·0	728·0, 843·0	767·0	722·0, 851·0	1·0	–7·7, 4·9	0·083*
Urinary													
Iodine, ug/g	175·0	120·0, 216·0	294·0	207·0, 421·0	85·0	5·1, 162·0	180·5	140·0, 288·0	182·0	116·0, 344·0	–2·4	–23·4, 73·8	0·017
Li§, ug/g	25·0	18·0, 40·0	777·5	348·0, 1034·0	1679	855·9, 4242	29·0	22·0, 41·0	26·0	21·0, 30·0	–9·4	–25·8, 14·3	< 0·001
Se, ug/g	86·0	65·0, 116·0	119·0	99·0, 151·0	32·4	9·3, 54·7	92·0	74·0, 114·0	84·0	67·0, 112·0	–7·3	–21·2, 20·2	< 0·001

* *P* value for between group difference calculated using two-sample *t* test, all others Mann–Whitney test.

† Oregon participants only (*n* 37: 23 multinutrient, 14 placebo).

‡ Ohio participants only (*n* 38: 23 multinutrient, 15 placebo).

§ Results missing for fourteen urinary samples for lithium (*n* 55: 30 multinutrient, 25 placebo).

To assess for potential bias introduced by the site differences for V, Li and Fe, a sensitivity analysis examined each site separately for 8-week percent change for these three minerals. The results did not differ: Li and V 8-week percent change remained significantly different for both sites, while Fe 8-week percent change remained non-significant for both sites (online Supplementary Table 4). Thus, the sensitivity analysis generally confirmed the overall findings. Additionally, due to differences by sex at baseline in Cu, Cr and Ni and difference in male/female ratio between intervention arms, another sensitivity analysis examined each sex separately for these three minerals. Cr had a significant increase in 8-week percent change for multinutrient *v*. placebo for females (78·38 % (46·43 %, 238·10 %) *v*. −5·08 % (–40·28 %, 0·00 %), z = 2·94, *P* = 0·002), but not for males (–7·38 % (–52·76 %, −33·78 %) *v*. −30·35 % (–50·21 %, −35·70 %), z = 0·136, *P* = 0·901). Cu and Ni 8-week percent change remained non-significant for both sexes (online Supplementary Table 4). Again, the sensitivity analysis generally confirmed the overall findings.

### Baseline mineral concentrations as moderators and predictors

**Fig. 5.**
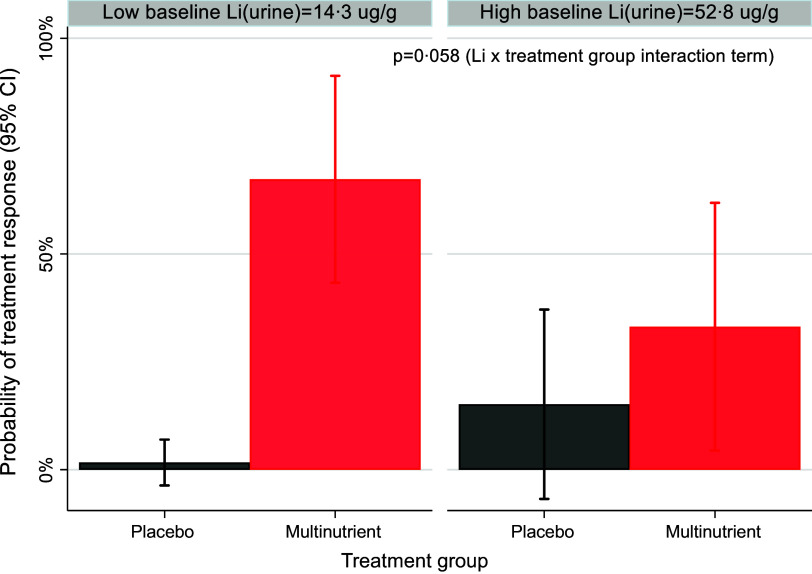
The moderating effect of baseline urinary Li concentration by treatment group on the probability of treatment response. Participants with lower baseline urinary Li levels (represented as 1 sd below the mean, 14·3 ug/g creatinine) were more likely to be responders than those with higher baseline levels (represented as 1 sd above the mean, 52·8 ug/g creatinine) in the multinutrient group.

**Fig. 6.**
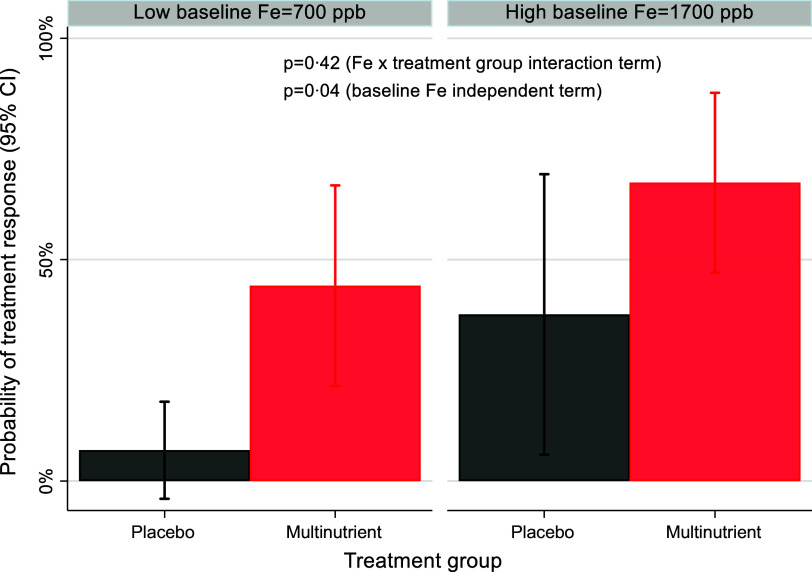
The independent predictor effect of baseline Fe level on the probability of treatment response. Participants with higher baseline plasma Fe levels (represented as 1 sd above the mean, ∼1700ppb) were more likely to be responders than participants with low baseline Fe (represented as 1 sd below the mean, ∼700ppb) in both treatment groups.

No baseline mineral concentration had a significant three-way interaction with site and intervention arm; therefore, site was handled only as a covariate in the moderation models, and moderation was tested as a two–way interaction of baseline mineral concentration × intervention arm. As shown in Table 3, baseline urinary Li concentration was not a significant moderator of treatment response, though the *P* value was trending (OR: 0·906; 95 % CI: 0·817, 1·003; z = −1·90; *P* = 0·058). Specifically, participants with lower baseline urinary Li levels were more likely to be responders than those with higher baseline levels in multinutrient group, while baseline levels did not affect response to placebo (Fig. 5). Additionally, baseline plasma Fe was a significant independent predictor of treatment response (OR: 1·002; 95 % CI: 1·000, 1·003; z = 2·10; *P* = 0·036). Participants with higher baseline Fe levels were more likely to be responders to both multinutrients and placebo (Fig. 6).

**Table 3.** Results of moderator analysis of clinical treatment response for each baseline mineral level (Odds ratios and 95 % confidence intervals)

		Moderator				Independent predictor			
		baseline × treatment group interaction				Baseline concentration			
	*n*	OR	95 % CI	z	*P*	OR	95 % CI	z	*P*
Plasma									
Boron*	37	0·923	0·775, 1·098	–0·91	0·365	1·019	0·945, 1·099	0·48	0·628
Cr	75	0·801	0·486, 1·319	–0·87	0·383	1·239	0·954, 1·608	1·61	0·108
Cu	75	1·004	0·996, 1·012	1·07	0·286	1·000	0·995, 1·005	0·01	0·991
Fe	75	0·999	0·996, 1·002	–0·81	0·421	1·002	1·000, 1·003	2·1	0·036
Li	75	0·287	0·031, 2·636	–1·1	0·270	1·976	0·647, 6·040	1·2	0·232
Mg (WB)*	37	1·016	0·569, 1·813	0·05	0·958	0·973	0·732, 1·294	–0·19	0·852
Mg (RBC)†	38	1·307	0·838, 2·037	1·18	0·238	0·959	0·768, 1·199	–0·36	0·716
Mn	75	3·040	0·012, 746·460	0·4	0·692	0·656	0·040, 10·620	–0·3	0·766
Mo	75	0·264	0·002, 29·155	–0·56	0·579	1·410	0·129, 15·401	0·28	0·778
Nickel	75	1·634	0·345, 7·741	0·62	0·536	0·815	0·375, 1·771	–0·52	0·605
Phosphorus	75	1·014	0·972, 1·057	0·64	0·520	1·006	0·976, 1·037	0·4	0·686
Se	75	1·021	0·968, 1·078	0·77	0·442	1·017	0·985, 1·050	1·04	0·296
Vanadium	75	0·812	0·494, 1·336	–0·82	0·413	1·198	0·906, 1·582	1·27	0·205
Zinc	75	1·003	0·994, 1·013	0·72	0·470	1·001	0·996, 1·006	0·5	0·617
Urine									
Iodine	69	0·993	0·982, 1·003	–1·32	0·186	1·003	0·997, 1·008	1·01	0·313
Li	55	0·906	0·817, 1·003	–1·9	0·058	1·012	0·960, 1·067	0·45	0·656
Se	69	0·984	0·942, 1·027	–0·75	0·454	1·000	0·978, 1·022	–0·03	0·975

* OR participants only.

† OH participants only.

### 8-week percent change in mineral concentrations as mediators

No 8-week percent change had a significant three-way interaction with site and intervention arm; therefore, site was handled merely as a covariate in the mediation models, and mediation was tested as a two–way interaction of 8-week percent change × intervention arm. The minerals with significant between-group 8-week percent change (B, Cr, Li, Mo, P, Se, V, I, Li(urine), Se(urine)) were evaluated as potential mediators of treatment response. As shown in Table 4, none of the mineral 8-week percent changes were significant mediators of treatment response.

**Table 4.** Results of mediator analysis for all minerals with significant between-group 8-week % change (Odds ratios and 95 % confidence intervals)

		8-week change × treatment group interaction			
Mineral	*n*	Odds ratio	95 % CI	z	*P*
Plasma					
Boron*	37	1·007	0·974, 1·042	0·42	0·678
Cr	75	1·039	0·994, 1·086	1·69	0·092
Li	75	1·003	0·992, 1·014	0·56	0·577
Mo	75	0·988	0·969, 1·008	–1·160	0·246
Phosphorus	75	1·081	0·934, 1·252	1·05	0·295
Se	75	0·905	0·775, 1·057	–1·26	0·208
Vanadium	*75*	1·020	0·987, 1·054	1·18	0·239
Urine					
Iodine	69	1·000	0·995, 1·004	–0·17	0·862
Li	55	1·001	0·996, 1·006	0·32	0·748
Se	69	1·001	0·963, 1·040	0·03	0·975

* OR participants only.

## Discussion

In this study, we explored the time trends of fourteen mineral concentrations in plasma and/or urine from an 8-week RCT of multinutrient supplementation compared with placebo. The finding of higher baseline plasma Cr in males compared with females contrasts with another study of children with ADHD, which found no significant differences in serum Cr in either sex between ADHD and non-ADHD groups^(30)^. Although the role of Cr in neurological function is not well understood, its role in glucose metabolism and improving insulin sensitivity in the hypothalamus is hypothesised to potentially lead to increased synthesis of serotonin and catecholamines^(31)^.

Following 8 weeks of multinutrients compared with placebo, study participants had significantly increased levels of B, I (urine), Li (plasma and urine), Mo, Se (plasma and urine), V, and a trend for Zn, and a decrease in P. There is limited literature on mineral concentrations in children with ADHD after multinutrients, especially measuring these lesser studied minerals; therefore, results will be compared with studies of adults with ADHD and children with autism spectrum disorder, a similar and often co-occurring neurodevelopmental disorder. In a three-month RCT of multinutrients in fifty-three autistic children that measured mineral status in serum, whole blood, and RBC, and iodine in urine, pre-and post-treatment, detected increases in I (urine), Li (WB), Mo (WB), Se (WB), and a decrease in P (RBC) in the multinutrient group. However, the study contrarily found significant decreases in B (RBC) and Se (RBC) and no change in P (serum) and V (RBC)^(32)^.

Contrary to our hypothesis, several plasma mineral levels did not change after 8-weeks of supplementation (Cu, Fe, Mg (WB and RBC), Mn, Ni), which generally aligns with other published studies on multinutrients use in adults with ADHD and children with autism. A study of adults with ADHD found no change in serum ferritin, serum Fe, plasma Zn or plasma Cu after 8- or 10-weeks of multinutrients treatment^(33)^. Similarly, in the study of fifty-three autistic children that received 3 months of multinutrients, there was no significant change in Cu (WB), Cu (RBC), Cr (RBC), Fe (serum), Mg (serum), Mn (RBC), Zn (WB) and Zn (RBC) post-treatment, although there were increases in Mg (WB), Mn (WB) and decreases in Fe (RBC) and Mg (RBC)^(32)^. There is additional evidence of this effect from studies in adults without ADHD. A large observational study of older adults using NHANES data assessed the contribution of multivitamin/mineral (MVM) use to levels of nutrient biomarkers; both sporadic (1–15 d/month) and regular (>= 16 d/month) MVM users exhibited higher levels of serum Se and urinary I, but MVM use did not affect serum levels of Cu, Fe or Zn^(34)^. Hypotheses for lack of change in some minerals may be competition for absorption, or homoeostatic mechanisms in the body^(35,36)^. This finding is significant because it suggests that supplementation with a broad range of minerals levels may modulate the risk of over-exposure that could occur with single mineral supplementation of some minerals of potential concern in ADHD (i.e. excess Cu, Fe, Mn, etc.)^(37)^.

The large change in Li concentrations after supplementation in both the plasma and urine in the multinutrient group is noteworthy. Baseline urinary Li levels of the participants ranged from 12 to 117 µg/g Cr (median: 27·0, interquartile range: 21·0, 41·0), within reference ranges for adults 10–218 µg/g Cr (pediatric reference ranges not established)^(38)^. The amount of Li in the urine can be an indicator of the supply of the element, as about 97 % of oral intake is excreted by the kidneys within 24 h^(39)^. The primary sources of Li intake are cereal grains, nuts, seeds and vegetables, with additional contribution from drinking water and meat, with estimated daily intake of a 70 kg adult ranging from 0·65 to 3·1 mg^(40)^. The Li dosage in the multinutrient formula, 0·75–1 mg, is much lower than pharmaceutical doses (e.g. 300–1200 mg/d) used for children 12 years and older with bipolar disorder^(41)^.

The trend for baseline urinary Li levels as a potential moderator of treatment response may present a promising prospect for a non-invasive biomarker identifying which children may benefit from this intervention. Li at pharmacological doses is an established treatment for bipolar disorder due to its mood stabilising and anti-suicide effects^(42)^. Additionally, Li in combination with first-line pharmacotherapy for ADHD has been proposed as treatment for aggression in youth aged 5–20 years with ADHD^(43)^. Li has several potential biological effects that could impact children with ADHD and emotional dysregulation. Although there is little research in children, there is some evidence that low-dose Li (i.e. serum levels <= 0·6 mmol/l) may attenuate cognitive decline in adults and be effective as an adjuvant therapy for depression and mania with an improved safety profile compared with standard pharmaceutical doses of Li^(42)^. One potential physiological effect of Li is competition with Na and Mg ions due to their similar atomic size; this may lead to inhibition of enzymes, which affects the synthesis and release of neurotransmitters^(39)^. It is conceivable that people with bipolar and other mood disorders have a genetically higher need for Li to regulate mood levels, requiring ‘therapeutic doses,’ but that some other disorders, such as ADHD, especially with emotional dysregulation, share partial genetic disposition and need Li at slightly higher levels than in the usual diet but not ‘therapeutic doses.’ Alternatively, emotional dysregulation may be a consequence of lower Li intake and lower physiologic status generally in the presence or absence of the other manifestations of ADHD. Further study is required to elucidate these relationships.

The lack of other minerals moderating treatment response aligns with a previous study which found that none of the pre-treatment serum mineral levels (ferritin, Fe, Zn, Cu, Ca, and Mg) were associated with treatment response^(44)^. Additionally, our finding that greater baseline Fe levels are an independent predictor of response replicates another multinutrient study on adults with ADHD, which found that greater ferritin at baseline predicted ADHD responders^(45)^. The association of high Fe with treatment response was unexpected and could be a type 1 error or may have a nonparadoxical explanation: the amount of Fe in the supplement was merely at recommended intake level (i.e. RDA level), not a therapeutic level, in contrast to most of the other multinutrients. If a certain threshold of body Fe stores is needed for the other nutrients to work, and if body stores of participants are generally low, those with higher Fe would be more likely to have their total Fe body stores raised to a therapeutic level by this RDA increment. Such an explanation would be consistent with the broad-spectrum nutrient hypothesis that nutrients work together in a synergistic manner, and insufficiency or relative insufficiency of any one nutrient affects the balance of others^(37,46,47)^. This deserves further exploration in future studies.

Our finding that no mineral change mediated treatment response is in line with a previous study that evaluated change in serum ferritin, serum Fe, plasma Zn, plasma Cu against seven outcomes in children and adults with ADHD, which found only that a decrease in ferritin and an increase in Cu were weakly associated with one of the six outcomes^(33)^. The lack of significant mediation and moderation in this analysis results in several potential hypotheses: 1. The other vitamins contained in the formula may more strongly affect treatment response (though these have not yet been evaluated); 2. Mineral levels measured in plasma may not reflect levels in the central nervous system involved in ADHD; and 3. There may be multiple modes of action of multinutrients that cumulatively lead to improved ADHD outcomes leading to small effect sizes which are difficult to detect in this sample.

### Strengths and limitations

A strength of this analysis is the use of the placebo-control condition provided by the design of the RCT to attribute effects of the 8-week change and mediation and moderation directly to the multinutrient intervention. Measurement of the plasma and/or urine levels of fourteen minerals contained in the supplement, including trace minerals rarely studied in children, provides a holistic view that encompasses the complex interactive effects of a multinutrient supplement.

There are several limitations in this study. The standard error associated with the plasma mineral measurements was large and led to wider confidence intervals, which occurred, in part, because of the site differences. The site differences in plasma concentrations could potentially be due to sample collection or storage differences between sites or regional differences in mineral exposures from soil conditions and water supplies^(48,49)^. This was partially accounted for by analysing percentage change in the mineral levels which diminished the site differences, including site as a covariate in regression models, and additionally performing sensitivity analyses based on site to verify main findings. We did not control for overall Type I error since these are exploratory and hypothesis-generating analyses. Plasma mineral levels may not reflect levels in the central nervous system involved in ADHD. Finally, although plasma and urine concentration are commonly studied and are standard measures for some nutrients (e.g. plasma Se and urinary iodine), they may not be best indicator of body mineral status for all minerals^(50)^.

### Clinical significance

In the era of personalised nutrition, research needs to determine the mechanisms by which nutrients influence health and disease. While this has been examined for a number of health conditions (e.g. cancer and cardiovascular disease), this is lacking in ADHD. Evaluating mineral biomarkers for a treatment that has shown benefit in two RCTs of children with ADHD is a first step to applying concepts of personalised nutrition. The use of a multinutrient supplement containing fourteen essential minerals by children with ADHD for 8 weeks showed significant behaviour improvements. The intervention also changed plasma levels of six out of thirteen measured minerals and urinary levels of three out of three measured minerals, demonstrating its physiological effects. Multinutrient supplementation may be an effective treatment for children with ADHD and emotional dysregulation regardless of their baseline plasma mineral levels; testing children’s plasma mineral levels may be unnecessary when considering this treatment option. In contrast, baseline urinary Li levels may have the potential to be a non-invasive biomarker of those who may be most likely to respond to treatment, though further studies are needed to replicate these findings, and to define the appropriate Li range.

### Implications for future research

Boron, Cr, I, Mo, P, Se, V and Li in particular should be considered in future hypotheses to identify biological mechanisms of action of multinutrients for ADHD and emotional dysregulation. Theoretically, increased Se could lead to improved antioxidant enzyme activity and improved response to oxidative stress, or increased Li may alter levels of neurotransmitters in the brain. Further studies are required to replicate the finding that baseline urinary Li levels may be an effective biomarker for predicting which children with ADHD may respond to treatment with multinutrients. Dietary changes to increase Li intake, such as increasing grain and vegetable consumption, could be explored as another potential complementary therapy to improve symptoms of ADHD and emotional dysregulation, with minimal side effects.

### Conclusions

The evaluation of mineral biomarkers to determine potential mechanisms by which nutrients influence ADHD is a first step towards applying a personalised nutrition approach to this treatment for ADHD. The detection of rarely-assessed essential and trace minerals in plasma, such as B, Li, Mo, V, indicates that plasma concentrations are sensitive measures of change in these minerals induced by multinutrient supplementation and identifies Li status as a mineral of interest in ADHD, warranting further research and confirmation. Other baseline plasma and urinary mineral levels and their changes after 8 weeks did not moderate or mediate treatment response, suggesting that pre-treatment plasma mineral concentrations may not predict response. However, the failure to find moderators or mediators may be a type 2 error due to insufficient power.

## Supporting information

Robinette et al. supplementary material Robinette et al. supplementary material

## Supplementary material

For supplementary material/s referred to in this article, please visit https://doi.org/10.1017/S0007114524001132
