# Supplementary material for: Evaluating mineral biomarkers as mediators and moderators of behavioural improvements in a randomised controlled trial of multinutrients for children with attention-deficit/hyperactivity disorder
Source: Br J Nutr. 2024 May 31;132(3):315–29. doi: 10.1017/S0007114524001132 (PMC11473203; doi:10.1017/S0007114524001132)
Supplement: Robinette et al. supplementary material [file S0007114524001132sup001.docx]

**Evaluating Mineral Biomarkers as Mediators and Moderators of Behavioural Improvements in a Randomized Controlled Trial of Multinutrients for Children with ADHD**

**Supplemental materials**

Ingredient label for multinutrient supplement


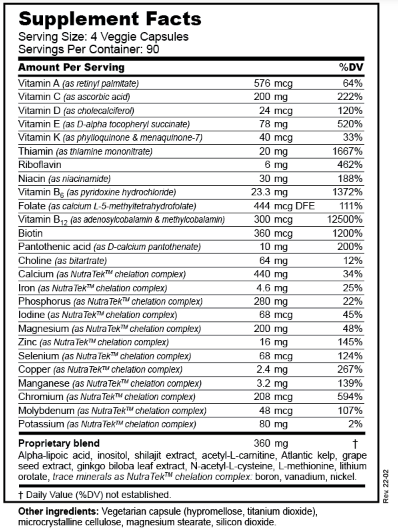


**Plasma Mineral Concentrations**

Mean and range of CoV across all samples for each mineral

|  | Mean CoV | Range |
| --- | --- | --- |
| Boron | 5.3% | 1.4-15.5% |
| Chromium | 0.1% | 0.0-0.8% |
| Copper | 8.3% | 4.0-16.0% |
| Iron | 19.5% | 6.3-76.2% |
| Lithium | 0.9% | 0.0-8.1% |
| Magnesium | 1.0% | 0.4-9.2% |
| Manganese | 0.0% | 0.0-0.4% |
| Molybdenum | 0.0% | 0.0-0.1% |
| Nickel | 0.1% | 0.0-2.3% |
| Phosphorus | 9.7% | 3.7-22.0% |
| Selenium | 2.0% | 1.2-3.0% |
| Vanadium | 0.1% | 0.0-0.8% |
| Zinc | 12.8% | 8.4-18.9% |

**Urinary Mineral Concentrations**

Intra- and inter-assay coefficient of variances

Source: ZRT Laboratories Dried Urine Test Specifications

|  | Intra-assay CoV | Inter-assay CoV |
| --- | --- | --- |
| Iodine | 3.5% | 4.1% |
| Lithium | 2.4% | 9.9% |
| Selenium | 5.1% | 9.9% |

Supplemental Table 1. Differences between site and sex for baseline mineral concentrations.

|  | **Sex** | | | **Site** | | |
| --- | --- | --- | --- | --- | --- | --- |
|  | **male (n=53)** | **female (n=22)** | **p-value^1^** | **Oregon (n=37)** | **Ohio (n=38)** | **p-value^1^** |
| ***Baseline blood mineral levels*** |  |  |  |  |  |  |
| Boron ^2^, ug/L, mean (SD) | 29.54 (9.53) | 27.50 (14.02) | 0.605 | 28.88 (11.03) | NA | NA |
| Chromium, ug/L, median (IQR) | 1.24 (0.73-2.39) | 0.66 (0.52-0.97) | 0.001 | 0.69 (0.52-0.96) | 1.59 (0.97-3.11) | 0.000 |
| Copper, ug/L, mean (SD) | 931.8 (161.1) | 839.0 (144.6) | 0.022 | 843.27 (178.70) | 964.32 (116.09) | 0.001 |
| Iron, ug/L, median (IQR) | 1,233 (845-1,402) | 1,155 (834-1,375) | 0.538 | 1,305 (1,124-1,502) | 990 (776-1,300) | 0.003 |
| Lithium, ug/L, median (IQR) | 1.28 (1.03-1.67) | 1.19 (0.83-1.44) | 0.312 | 1.30 (1.19-1.96) | 1.04 (0.79-1.43) | 0.000 |
| Magnesium (WB) ^2^, mg/L, median (IQR) | 31.38 (29.71-33.80) | 30.18 (28.81-33.16) | 0.36 | 31.27 (29.33-33.64) | NA | NA |
| Magnesium (RBC) ^3^, mg/L, mean (SD) | 45.23 (5.25) | 46.01 (1.86) | 0.651 | NA | 45.43 (4.59) | NA |
| Manganese, ug/L, median (IQR) | 0.63 (0.45-0.83) | 0.65 (0.49-0.77) | 0.784 | 0.77 (0.66-0.89) | 0.49 (0.38-0.61) | 0.000 |
| Molybdenum, ug/L, mean (SD) | 1.21 (0.28) | 1.09 (0.23) | 0.081 | 1.18 (0.27) | 1.17 (0.28) | 0.896 |
| Nickel, ug/L, median (IQR) | 1.49 (1.01-2.01) | 0.97 (0.56-1.42) | 0.012 | 0.73 (0.53-1.85) | 1.49 (1.17-1.84) | 0.003 |
| Phosphorus, mg/L, median (IQR) | 154 (141-186) | 149 (133-181) | 0.236 | 140 (133-145) | 184 (163-192) | 0.000 |
| Selenium, ug/L, mean (SD) | 194.08 (23.53) | 186.28 (22.31) | 0.189 | 178.54 (18.57) | 204.69 (20.10) | 0.000 |
| Vanadium, ug/L, median (IQR) | 1.07 (0.22-2.01) | 0.25 (0.19-1.20) | 0.070 | 0.19 (0.16-0.24) | 1.82 (1.26-2.96) | 0.000 |
| Zinc, ug/L, median (IQR) | 777 (729-854) | 770 (700-815) | 0.365 | 736 (696-791) | 828 (761-892) | 0.000 |
| ***Baseline urinary mineral levels*** | **male (n=48)** | **female (n=21)** | **p-value^1^** | **Oregon (n=36)** | **Ohio (n=33)** | **p-value^1^** |
| Iodine, ug/g, median (IQR) | 175 (124-259) | 186 (125-239) | 0.734 | 171 (123-257) | 197 (126-247) | 0.7047 |
| Lithium ^4^, ug/g, median (IQR) | 27 (21-44) | 27 (23-32) | 0.395 | 27 (22-43) | 27 (18-40) | 0.396 |
| Selenium, ug/g, median (IQR) | 88 (69-116) | 88 (76-111) | 0.681 | 83 (65-118) | 99 (74-116) | 0.3587 |

1 Two-sample t-test for parametric variables, Mann-Whitney for non-parametric

2 Oregon participants only (n=37: 25 male, 12 female)

3 Ohio participants only (n=38: 28 male, 10 female)

4 results missing for 14 urinary samples for lithium (n=55: 37 male, 18 female; 36 Oregon, 19 Ohio)

Supplemental Table 2. Correlations between baseline mineral concentrations and sociodemographic, biometric, and baseline nutrient intake [data is presented as correlation coefficient (p-value)].

|  |  |  | **Estimated baseline nutrient intake from Vioscreen^TM^ food frequency questionnaire** | | | | | | | | |
| --- | --- | --- | --- | --- | --- | --- | --- | --- | --- | --- | --- |
| **Baseline mineral levels** | **Child age (years)** | **BMI (kg/m2)** | **Ca** | **Cu** | **Fe** | **Mg** | **Mn** | **P** | **K** | **Se** | **Zn** |
| *Blood* |  |  |  |  |  |  |  |  |  |  |  |
| Boron ^1,^ ug/L | -0.24 (0.17) | -0.05 (0.77) |  |  |  |  |  |  |  |  |  |
| Chromium, ug/L | 0.04 (0.74) | -0.04 (0.71) |  |  |  |  |  |  |  |  |  |
| Copper, ug/L | -0.20 (0.09) | 0.21 (0.07) |  | 0.08 (0.48) |  |  |  |  |  |  |  |
| Iron, ug/L | -0.04 (0.74) | -0.02 (0.88) |  |  | 0.17 (0.15) |  |  |  |  |  |  |
| Lithium, ug/L | 0.05 (0.67) | -0.29 (0.01) |  |  |  |  |  |  |  |  |  |
| Magnesium (WB) ^1^, mg/L | 0.27 (0.11) | 0.28 (0.10) |  |  |  | 0.20 (0.25) |  |  |  |  |  |
| Magnesium (RBC) ^2^, mg/L | 0.11 (0.53) | 0.21 (0.20) |  |  |  | 0.29 (0.08) |  |  |  |  |  |
| Manganese, ug/L | 0.06 (0.65) | -0.24 (0.04) |  |  |  |  | -0.06 (0.59) |  |  |  |  |
| Molybdenum, ug/L | -0.25 (0.04) | -0.25 (0.04) |  |  |  |  |  |  |  |  |  |
| Nickel, ug/L | 0.04 (0.75) | -0.12 (0.32) |  |  |  |  |  |  |  |  |  |
| Phosphorus, mg/L, | -0.03 (0.81) | -0.02 (0.88) |  |  |  |  |  | 0.13 (0.27) |  |  |  |
| Selenium, ug/L | -0.08 (0.49) | -0.03 (0.81) |  |  |  |  |  |  |  | 0.06 (0.59) |  |
| Vanadium, ug/L | 0.00 (0.99) | -0.01 (0.93) |  |  |  |  |  |  |  |  |  |
| Zinc, ug/L | -0.04 (0.74) | -0.04 (0.71) |  |  |  |  |  |  |  |  | 0.02 (0.89) |
| *Urine* |  |  |  |  |  |  |  |  |  |  |  |
| Iodine, ug/g | -0.30 (0.01) | -0.16 (0.12) |  |  |  |  |  |  |  |  |  |
| Lithium ^3^, ug/g | -0.23 (0.09) | -0.34 (0.01) |  |  |  |  |  |  |  |  |  |
| Selenium, ug/g | -0.40 (0.001) | -0.12 (0.32) |  |  |  |  |  |  |  | 0.23 (0.06) |  |

1 Oregon participants only (n=37: 25 male, 12 female)

2 Ohio participants only (n=38: 28 male, 10 female)

3 results missing for 14 urinary samples for lithium (n=55: 37 male, 18 female; 36 Oregon, 19 Ohio)

**Supplemental table 3. 8-week % change compared between sites.**

| **Mineral** | **Oregon** | | **Ohio** | | **p-value** |
| --- | --- | --- | --- | --- | --- |
|  | **mean (SD)** | **median (IQR)** | **mean (SD)** | **median (IQR)** |  |
| ***Blood*** |  |  |  |  |  |
| Boron | 71.69 (96.99) | 50.00 (11.43-111.11) | NA | NA | NA |
| Chromium | 55.96 (170.91) | 0.00 (-39.44-88.46) | -8.63 (59.75) | -18.74 (-52.76-15.85) | 0.075 |
| Copper | 4.17 (20.82) | 0.13 (-4.80-8.23) | 1.39 (14.37) | -2.79 (-7.70-8.54) | 0.357 |
| Iron | -7.05 (39.57) | -15.78 (-28.17-20.33) | 10.97 (43.27) | 6.47 (-15.12-36.27) | 0.041 |
| Lithium | 633 (816) | 480 (2-1,040) | 1,356 (1,401) | 1,264 (29-2,407) | 0.013 |
| Magnesium (WB) | 1.97 (13.40) | -0.37 (-3.85-9.78) | NA | NA | NA |
| Magnesium (RBC) | NA | NA | 1.12 (12.12) | 2.70 (-3.33-9.96) | NA |
| Manganese | 39.26 (106.46) | 13.23 (-17.24-37.96) | 55.58 (200.31) | 2.75 (-20.56-36.34) | 0.510 |
| Molybdenum | 32.34 (56.76) | 10.34 (-4.26-53.52) | 44.21 (94.33) | 17.50 (-15.56-53.04) | 0.862 |
| Nickel | 260.28 (779.65) | -7.69 (-45.56-77.16) | 27.30 (204.13) | -17.58 (-36.08-9.17) | 0.445 |
| Phosphorus | 0.03 (8.17) | 0.00 (-4.96-3.57) | -2.44 (9.82) | -2.79 (-8.72-4.07) | 0.240^1^ |
| Selenium | 1.79 (8.62) | 1.00 (-3.25-6.98) | 1.20 (8.43) | 1.42 (-3.96-6.74) | 0.763^1^ |
| Vanadium | 324.34 (485.69) | 62.82 (23.03-434.05) | 44.65 (85.95) | 29.47 (-10.00-77.36) | 0.006 |
| Zinc | 3.73 (11.58) | 4.20 (-4.74-9.05) | 1.17 (12.56) | 0.49 (-6.18-7.02) | 0.363^1^ |
| ***Urine*** |  |  |  |  |  |
| Iodine | 63.66 (131.82) | 22.96 (-19.23-123.05) | 160.88 (371.35) | 58.45 (-7.38-154.59) | 0.373 |
| Lithium | 1,208.48 (1,869.80) | 118.05 (-19.26-1,518.31) | 1,807.12 (1,937.37) | 1,375.00 (14.29-2,887.50) | 0.065 |
| Selenium | 27.76 (43.82) | 24.06 (-7.86-51.81) | 15.66 (45.87) | 9.26 (-12.50-30.66) | 0.155 |

^1^p-value for between group difference calculated using two-sample t-test, all others Mann-Whitney U-test

Supplemental Table 4. Sensitivity analysis of 8-week % change by site for minerals with significant site differences in 8-week % change (V, Li, Fe) and those with sex difference in baseline mineral levels (Cu, Cr, Ni). Values reported are median (IQR) of the 8-week % change for each subgroup.

|  | **Sensitivity Analysis for each Site** | | | | | | | | | | | |
| --- | --- | --- | --- | --- | --- | --- | --- | --- | --- | --- | --- | --- |
|  | **Oregon only** | | | | | | **Ohio only** | | | | | |
|  | **multinutrient** | | **placebo** | |  |  | **multinutrient** | | **placebo** | |  |  |
|  | **n** | **median (IQR)** | **n** | **median (IQR)** | **z** | **p-value** | **n** | **median (IQR)** | **n** | **median (IQR)** | **z** | **p-value** |
| Iron | 23 | -17.44  (-28.28-20.33) | 14 | -13.44  (-25.36-23.39) | -0.72 | 0.486 | 23 | -8.35  (-27.52-22.57) | 15 | 22.06  (-4.54-38.05) | -1.389 | 0.172 |
| Lithium | 23 | 808  (527-1,319) | 14 | -2  (-23-27) | 3.977 | 0.000 | 23 | 1,760  (1,300-2,875) | 15 | 25  (-5-67) | 4.285 | 0.000 |
| Vanadium | 23 | 313.1  (140.7-795.0) | 14 | -1.3  (-28.7-27.7) | 4.917 | 0.000 | 23 | 50.7  (23.5-136.1) | 15 | -9.4  (-37.7-8.1) | 3.181 | 0.001 |
|  | **Sensitivity Analysis for each Sex** | | | | | | | | | | | |
|  | **Males only** | | | | | | **Females Only** | | | | | |
|  | **multinutrient** | | **placebo** | |  |  | **multinutrient** | | **placebo** | |  |  |
|  | **n** | **median (IQR)** | **n** | **median (IQR)** | **z** | **p-value** | **n** | **median (IQR)** | **n** | **median (IQR)** | **z** | **p-value** |
| Chromium | 37 | -7.38  (-52.76-33.78) | 16 | -30.35  (-50.21-35.70) | 0.136 | 0.901 | 9 | 78.38  (46.43-238.10) | 13 | -5.08  (-40.28-0.00) | 2.94 | 0.002 |
| Copper | 37 | -2.73  (-9.95-6.06) | 16 | -1.23  (-6.19-11.70) | -1.085 | 0.286 | 9 | -0.99  (-1.55-17.74) | 13 | 1.52  (-3.32-9.84) | -0.1 | 0.935 |
| Nickel | 37 | -26.85  (-60.24-54.55) | 16 | -14.49  (-36.94-18.37) | -1.066 | 0.294 | 9 | -2.11  (-17.81-67.86) | 13 | -3.31  (-33.33-20.45) | 0.968 | 0.358 |
